# Dynamic Video Assessment of Axial Postural Abnormalities in Parkinson's Disease: A Pilot Study

**DOI:** 10.1002/mdc3.14329

**Published:** 2025-01-29

**Authors:** Carlo Alberto Artusi, Christian Geroin, Clarissa Pandino, Serena Camozzi, Stefano Aldegheri, Leonardo Lopiano, Michele Tinazzi, Nicola Bombieri

**Affiliations:** ^1^ Department of Neuroscience “Rita Levi Montalcini” University of Turin Turin Italy; ^2^ Neurology 2 Unit, Department of Neuroscience A.O.U. Città della Salute e della Scienza di Torino Torino Italy; ^3^ Neurology Unit, Movement Disorders Division, Department of Neurosciences Biomedicine and Movement Sciences University of Verona Verona Italy; ^4^ Department of Surgery, Dentistry, Paediatrics and Gynaecology University of Verona Verona Italy; ^5^ Department of Computer Science University of Verona Verona Italy

**Keywords:** axial postural abnormalities, Pisa syndrome, camptocormia, Parkinson's disease, objective assessment

## Abstract

**Background:**

Axial postural abnormalities (APAs) are frequent and disabling axial symptoms of Parkinson's disease (PD). Image‐based measurement is considered the gold standard but may not accurately detect the true severity of APAs because these symptoms can appear or get worse under dynamic conditions.

**Objective:**

The aim was to evaluate quantitative changes in APAs degree during prolonged standing and walking in both single‐ and dual‐task conditions (motor + cognitive).

**Methods:**

We measured the degree of anterior and lateral trunk flexion (LTF) of 16 PD patients using AutoPosturePD during 4 tasks of 3 min each: (1) standing in place in a quiet condition, (2) standing in place while reading, (3) walking without performing other tasks, and (4) walking performing a cognitive task.

**Results:**

During prolonged standing, we found a significant LTF worsening under both single‐ and dual‐task conditions over time (*P*: 0.010 and 0.018); anterior trunk flexion (ATF) with thoracic and lumbar fulcrum showed a significant worsening only under dual‐task conditions (*P* < 0.05). All trunk flexion angles were higher during dual task compared to single task, and the difference in dual task was already statistically significant after 1 min. During walking, only ATF with lumbar fulcrum showed a significant worsening (*P* < 0.05), observed in dual task already after 1 min.

**Conclusions:**

Our pilot study suggests that one minute standing while reading may be sufficient to obtain a more reliable measure of the severity of LTF and ATF, with an expected change of ~ 7° for LTF and ATF with thoracic fulcrum and 11° for ATF with lumbar fulcrum.

Axial postural abnormalities (APAs) are frequent, disabling, and often painful motor complications of Parkinson's disease (PD).^1^ They consist of abnormal trunk flexion appearing in the upright position, interfering with dexterity and daily‐life activities.^2^, ^3^ It has been estimated that over 20% of PD patients may develop one or more APAs such as camptocormia and Pisa syndrome or their milder forms.^1^, ^2^, ^3^ The heterogeneity in their classification and the assessment methods has contributed to the elusive epidemiology, pathophysiology, and evidence regarding the effect of therapeutic strategies, including dopaminergic therapy, physical therapy, botulinum toxin, and deep brain stimulation.^3^ Recently, a task force of the International Parkinson and Movement Disorders Society (MDS) developed a consensus for harmonizing nosology, classification, and cutoffs defining APAs in PD.^2^ The consensus relied on a photo‐based, semiautomated method, namely NeuroPostureApp, for measuring trunk flexion angles in PD patients using image analysis of individuals in a static standing position.^2^, ^4^ Subsequently, a new software, AutoPosturePD, was developed to enable the automatic capture of trunk flexion angles, simplifying the clinical evaluation of static posture.^5^, ^6^

However, these image‐based measurements may not accurately detect the true degree angle because APAs might appear or get worse under dynamic conditions, such as during walking or under dual‐task situations, resulting in different degrees of severity when compared with the static assessment.^7^, ^8^

APAs interfere with numerous patient's everyday activities that may require the concurrent execution of motor and cognitive tasks, such as talking while walking. Previous studies have demonstrated that these tasks contend for similar cognitive resources and may lead to a decreased performance in one or both tasks, also known as “cognitive‐motor interference” and quantified experimentally by the dual‐task cost.^7^, ^9^, ^10^, ^11^ When complex tasks occur, healthy subjects prioritize the motor component over the cognitive one, namely “posture first” strategy, to preserve their position.^12^ PD patients are unable to properly reallocate attentional resources, prioritizing posture, because of difficulties in performing 2 tasks simultaneously, which may impair the involuntary ability to keep their trunk straight. The consequences are limitations in mobility, reduced autonomy in real‐life settings, and increased risk of falling. Therefore, the evaluation of APAs during walking and dual‐task conditions is crucial for detecting the true degree of APAs.

To evaluate APAs in these conditions, we upgraded the AutoPosturePD software and performed an exploratory study using the automatic video analysis of APAs according to MDS Task Force–proposed criteria^2^ during standing and walking in both single‐ and dual‐task conditions. Our hypothesis is that APAs in PD patients may worsen under different and prolonged dynamic/dual‐task conditions and that these differences may be captured by a simple, automated video analysis with possible relevant implications from research and clinical standpoints.

## Patients and Methods

### Subjects

Sixteen PD patients with different degrees of anterior trunk flexion (ATF) or lateral trunk flexion (LTF) while standing were consecutively enrolled at the Movement Disorders Center of the University Hospital of Verona, Italy. Inclusion criteria were clinically established PD according to the 2015 Movement Disorder Society diagnostic criteria^13^; presence of isolated or combined forms of camptocormia with thoracic fulcrum, lumbar fulcrum, or Pisa syndrome or milder forms according to consensus‐based criteria^2^; modified Hoehn & Yahr stage 2 or 3; and ability to walk for 3 consecutive minutes unassisted in the *on* medication state (crutches or canes were allowed). Exclusion criteria were patients with concomitant diseases other than PD affecting gait and posture, dementia or severe depression, psychiatric comorbidities, diagnosis of atypical parkinsonism (ie, multiple system atrophy, progressive supranuclear palsy, and cortico‐basal syndrome), and under treatment with medications possibly causing APAs (neuroleptics other than clozapine or quetiapine and antiemetics, except for domperidone) in the 6 months prior to enrollment; history of exposure to antipsychotics, valproate, and anticholinergics; and history of other neurological diseases, orthopedic diseases affecting gait or posture, and major spine surgery. Clinical assessment and video analysis were performed when participants were in the *on* medication state to minimize variability due to motor fluctuations. We excluded patients with moderate–severe dyskinesia in the *on* medication state to minimize the risk of inaccurate posture analysis in dynamic upright positions. All participants provided informed consent to participate in this study, which was approved by the local Ethics Committee and conducted in accordance with the Declaration of Helsinki.

### General Assessment

The following clinical and demographic characteristics were collected: sex (male, female); age (years); disease duration (since PD diagnosis, years); modified Hoehn & Yahr scale; the MDS Unified Parkinson's Disease Rating Scale (MDS‐UPDRS), Part III; PD motor phenotype (postural instability/gait disorder [PIGD], tremor dominant, or indeterminate);^14^ the type, duration, and degree of camptocormia; Pisa syndrome or milder forms such as ATF and LTF according to MDS criteria^2^; the type of walking aids; the Mini Balance Evaluation System Test (Mini‐BESTest) score; and the ongoing dopaminergic therapy.

### Postural Analysis

**FIG. 1:**
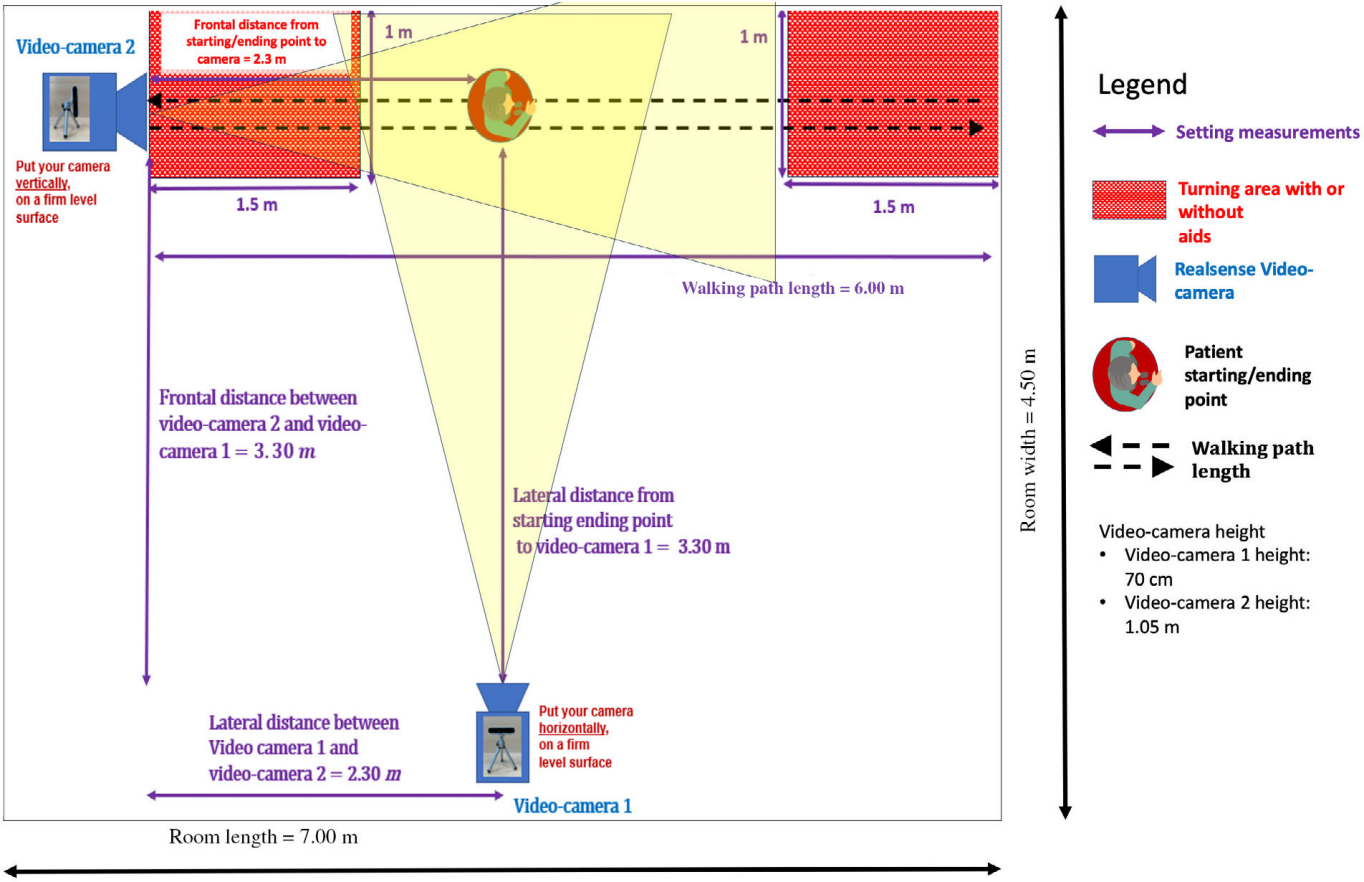
Setup for recording patient videos.

Patients were assessed during standing and walking tasks by a continuous video recording using 2 depth cameras (Realsense D415, Intel, Santa Clara, CA, USA). Patients' posture was assessed using an upgraded version of AutoPosturePD software^5^ for postural angle calculation, which automatically assesses the degrees of trunk flexion from RGB‐D cameras following the MDS Task Force criteria^2^ during dynamic conditions. The degrees of trunk flexion were automatically calculated in real time showing posture variations in degrees during prolonged standing and during walking. The angle of ATF with thoracic fulcrum, the angle of ATF with lumbar fulcrum, and the angle of LTF were calculated according to validated criteria.^2^ The experimental setup is shown in Figure 1.

Patients were asked to perform 4 tasks of 3 min each: (1) comfortable standing in place in a quiet condition (standing single task—s.standing), (2) comfortable standing in place while reading a text (standing dual task—d.standing), (3) walking back and forth without speaking or performing other tasks (walking single task—s.walking), and (4) walking back and forth counting back aloud from 100 to 0, subtracting 7 (walking dual task—d.walking). During dual‐task conditions, participants were asked to focus their attention primarily on the countdown, aiming for accuracy in the cognitive task. Whenever a mistake was made, the experimenter provided the correct number, prompting the participant to resume the subtraction from that point. Videos were taken during the entire time of the tasks. During standing tasks, patients were instructed to look forward without exaggerated head movements. During walking tasks, patients were required to walk straight at a self‐selected comfortable speed in the middle of a lane. For each task, patients were assessed in their underwear or (in case of impossibility of taking off their clothes), with a tight‐fitting jumpsuit to allow a more precise angle calculation from the AutoPosturePD software analysis. The same sequence of tasks was used for each patient, with an interval of at least 5 min between tasks. More details on setting, tasks, and angle measurements are provided in Supplementary Material S1.

### Statistical Analysis

Descriptive statistics with median, range, and frequency were used for continuous and categorical variables. The following data were extracted from the continuously recorded posture angle values by the software: the median value of the postural angle during standing tasks recorded over a 30‐s interval and the median value of the postural angle in walking tasks recorded during each cycle of the 6‐m path completed by the patient. Therefore, we used for each patient the values of postural angles as the median values of a 5‐s window obtained at baseline (T0) and at 1 min (T1), 2 min (T2), and 3 min (T3) for the analyses. The rationale for using a time window rather than a single‐point measurement was 2‐fold: first, to compensate for potential measurement jitter, and second, to mitigate temporary movements due to the resetting of the patients' posture.

For each task, the degrees of trunk flexion were analyzed over time comparing the changes at different time points using a one‐way repeated‐measures analysis of variance (ANOVA). To explore the differences between one task and another (single vs. dual task and standing vs. walking), we performed a two‐way repeated‐measures ANOVA test where we combined the independent variable of time (T0, T1, T2, T3) with the second independent variable represented by the patient's task (single task vs. dual task).

To evaluate the interference of the cognitive task on the degree of trunk flexion, we calculated the dual‐task cost using the following formula [4]: dual‐task cost (%) = −(DT – ST)/ST × 100, where DT is the performance at the dual task and ST the performance at the single task, using posture angles at T3. To facilitate the interpretation of the data, a minus symbol was added before the numerator subtraction. In this way, positive values represent more cost during the DT than the ST, indicating a worsening of posture driven by the cognitive task.^15^ Spearman's correlation between the change in ATF and LTF degrees between T0 and T3 and the Mini‐BESTest score and UPDRS, Part III, score was performed. The α level was set at 0.05. Statistical analysis was performed using IBM SPSS Statistics version 26.0 for Macintosh.

## Results

### Clinical Features of PD with APAs

The main demographic and clinical characteristics of the 16 PD patients included in the study are summarized in Table 1. All patients had 1 or more APAs. A total of 7 patients satisfied the criteria of camptocormia with lumbar fulcrum, with the degrees of trunk bending ranging from 31 to 62.7. ATF with lumbar fulcrum was found in 5 patients, with the degrees of trunk bending ranging from 19.2 to 28.6. A normal ATF with a lumbar fulcrum was found in 4 patients, with the degrees of trunk bending ranging from 1.9 to 13.8. Five patients satisfied the criteria of camptocormia with thoracic fulcrum, with the degrees of trunk bending ranging from 45.7 to 58.8. ATF with thoracic fulcrum was found in 9 patients, with the degrees of trunk bending ranging from 25 to 43.7. A normal ATF with thoracic fulcrum was found in 2 patients, with the degree of trunk bending of 12.6 and 18.3, respectively. One patient satisfied the criteria of Pisa syndrome, with 16.3 degrees of trunk bending. LTF was found in 6 patients, with the degrees of trunk bending ranging from 5 to 9.3. A normal LTF was found in 9 patients, with the degrees of trunk flexion ranging from 0.1 to 4.5.

**TABLE 1:** Clinical and demographical variables of patients with PD and axial postural abnormalities

Sex, age (y)	PD duration (y)	Modified H&Y score	MDS‐UPDRS, Part III, score	PD phenotype	APA type and degrees	APA duration (y)	Walking aid	Mini‐BEST test score	Dopaminergic treatment (LEDD)
M, 74	10	2	29	Indet	ATF lumbar fulcrum: 22.5° ATF thoracic fulcrum: 43.7° LTF (left): 3.9°	5	–	25	Levodopa 400 mg Rasagiline 1 mg Ropinirole PR 8 mg (660)
F, 77	10	3	34	Indet	CC lumbar fulcrum: 48.2° ATF thoracic fulcrum: 42.1° LTF (right): 1.7°	9	Crutch	7	Selegiline 10 mg Levodopa 600 mg (700)
F, 59	8	3	31	PIGD	ATF lumbar fulcrum: 1.9° ATF thoracic fulcrum: 18.3° PS (left): 16.3°	3	–	2	Levodopa 600 mg Melevodopa 100 mg Pramipexole PR 0.52 mg (775)
M, 72	10	2	12	PIGD	CC lumbar fulcrum: 38.9° ATF thoracic fulcrum: 43.4° LTF (right): 5°	3	–	23	Levodopa 600 mg (600)
F, 70	5	2	12	PIGD	CC lumbar fulcrum: 31° ATF thoracic fulcrum: 26° LTF (right): 6.5°	2	–	22	Levodopa 300 mg Selegiline 5 mg (350)
F, 78	6	3	25	Indet	CC lumbar fulcrum: 35.4° ATF thoracic fulcrum: 12.6° LTF (right): 5°	3	Walker	5	Levodopa 800 mg Pramipexole PR 1.05 mg (950)
M, 68	4	3	52	Indet	CC lumbar fulcrum: 42.8° CC thoracic fulcrum: 47.1° LTF (right) 1.7°	2	Walker	11	Levodopa 300 mg Selegiline 5 mg (350)
F, 58	3	2	9	PIGD	ATF lumbar fulcrum: 19.2° ATF thoracic fulcrum: 43.4° LTF (left): 4.2°	3	–	26	Pramipexole PR 1.05 mg Rasagiline 1 mg (250)
M, 69	14	3	24	Indet	ATF lumbar fulcrum: 28.6° ATF thoracic fulcrum: 35.4° LFT (right): 8°	7	Walker	26	Levodopa 800 mg Safinamide 50 mg Ropinirole PR 8 mg (1060)
M, 71	7	2	18	Indet	CC lumbar fulcrum: 40.4 CC thoracic fulcrum: 51.5° LTF (right): 4°	3	–	22	Levodopa 600 mg Safinamide 50 mg (700)
F, 79	4	2	7	PIGD	ATF lumbar fulcrum: 11.8° ATF thoracic fulcrum: 25° LTF (right): 9.3°	3	–	23	Levodopa 1000 mg Selegiline 10 mg Opicapone 50 mg (1600)
M, 72	4	2	8	PIGD	ATF lumbar fulcrum: 13.8 CC thoracic fulcrum: 51.1° LTF (right): 4°	2	–	25	Levodopa 400 mg Pramipexole PR 0.52 mg (475)
M, 57	1	2	9	PIGD	ATF lumbar fulcrum: 20.4° CC thoracic fulcrum: 45.7° LTF (right): 0.1°	1	–	26	Levodopa 400 mg Rasagiline 1 mg (500)
F, 83	5	3	21	Indet	ATF lumbar fulcrum: 12.3° ATF thoracic fulcrum: 25.7° LTF (left): 0.7°	1	–	17	Levodopa 300 mg Selegiline 5 mg (350)
F, 51	6	3	38	PIGD	CC lumbar fulcrum: 62.7° ATF thoracic fulcrum: 41° LTF (left): 4.5°	2	Walker	10	Levodopa 1000 mg Selegiline 10 mg Opicapone 50 mg (1600)
M, 80	3	3	25	PIGD	ATF with lumbar fulcrum: 25.2° CC with thoracic fulcrum: 58.8° LTF (right): 7.5°	2	Walker	17	Levodopa 400 mg Rasagiline 1 mg (500)

Abbreviations: PD, Parkinson's disease; H&Y, Hoehn & Yahr stage; MDS‐UPDRS, Part III, Movement Disorders Society Unified Parkinson's Disease Rating Scale, Part III; APAs, axial postural abnormalities; LEDD, levodopa equivalent daily dose; M, male; Indet, indeterminate phenotype; ATF, anterior trunk flexion; LTF, lateral trunk flexion; CC, camptocormia; PS, Pisa syndrome; PIGD, postural instability/gait disorder phenotype; F, female.

### Three‐Minute Standing (Single and Dual Tasks)

We found a significant worsening of LTF over time under single‐task (*P*: 0.010) and dual‐task (*P*: 0.018) conditions. The average flexion degree over 1, 2, and 3 min increased 1.4°, 2.1°, and 1.9°, respectively, in single task and 7.8°, 7.2°, and 6.6° in dual task. The difference was observed over time, with the highest difference in the median values at the second min for the single task and after 1 min for the dual task.

**FIG. 2:**
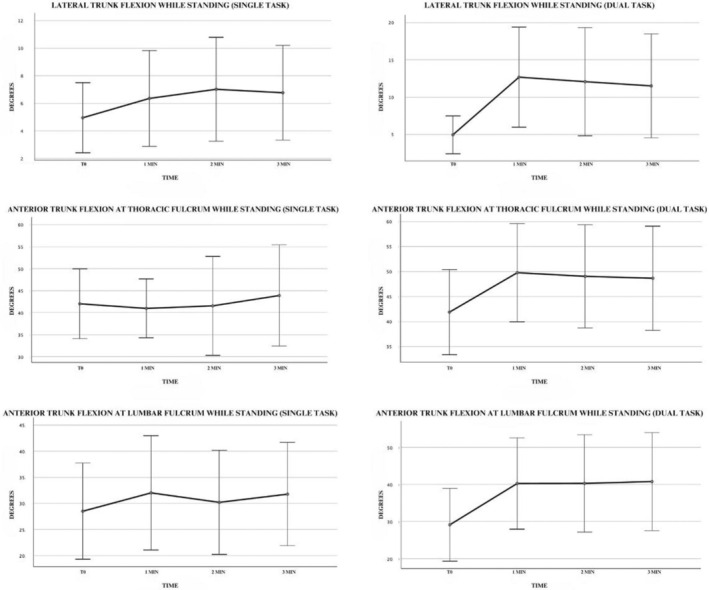
Charts representing the degree of trunk bending during single and dual tasks while standing.

ATF, both at the lumbar fulcrum and at the thoracic fulcrum, showed significant worsening over time only in dual‐task condition (*P*: <0.001 and *P*: 0.001, respectively), showing a statistically significant difference already at the first min (*P*: 0.006 and *P*: 0.011, respectively) (Table 2; Fig. 2).

**TABLE 2:** Comparison of trunk bending degrees over 3 min of static standing

Standing	Task	df	*F*	*P*‐value	Pairwise *P*‐value
Lateral trunk flexion/Pisa syndrome	Single task	1.800	5.904	**0.010**	T0 vs. 3 min, *P*: 0.054
	Dual task	1.836	4.925	**0.018**	T0 vs. 1 min, *P*: 0.073
Anterior trunk flexion/camptocormia with thoracic fulcrum	Single task	1.240	0.414	0.571	–
	Dual task	2.217	8.080	**0.001**	T0 vs. 1 min, *P*: **0.011** T0 vs. 2 min, *P*: **0.017** T0 vs. 3 min, *P*: **0.016**
Anterior trunk flexion/camptocormia with lumbar fulcrum	Single task	1.815	1.435	0.255	–
	Dual task	1.259	14.905	**<0.001**	T0 vs. 1 min, *P*: **0.006** T0 vs. 2 min, *P*: **0.007** T0 vs. 3 min, *P*: **0.009**

Values in bold font indicate statistically significant difference.

*P*‐values refer to one‐way ANOVA (analysis of variance) analysis, with post hoc pairwise comparisons.

The average flexion degree of ATF with thoracic fulcrum over 1, 2, and 3 min increased 7.4°, 6.7°, and 6.7°, respectively. The average flexion degree of ATF with lumbar fulcrum over 1, 2, and 3 min increased 10.8°, 10.9°, and 12.3° respectively.

### Three‐Minute Walking (Single and Dual Tasks)

We found a significant worsening of ATF at the lumbar fulcrum over time in both single‐task (*P*: 0.025) and dual‐task conditions (*P*: 0.004). The average flexion degree over 1, 2, and 3 min increased 4.5°, 5.1°, and 6.1° in single task and 5.9°, 6.5°, and 7.2° in dual task, respectively. The difference was observed over time, with the highest difference at the third min for the single and dual tasks, and a statistically significant difference already present after 1 min (*P*: 0.048) for the dual task.

**FIG. 3:**
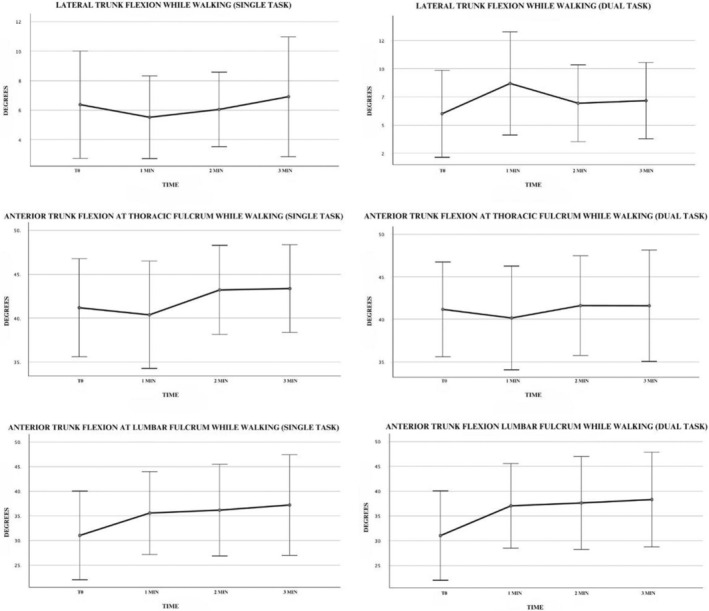
Charts representing the degree of trunk bending during single and dual tasks while walking.

No significant difference over time while walking was observed in the LTF nor in the ATF at the thoracic fulcrum (Table 3; Fig. 3).

**TABLE 3:** Comparison of trunk bending degrees over 3‐min of walking

Walking	Task	df	*F*	*P*‐value	Pairwise *P*‐value
Lateral trunk flexion/Pisa syndrome	Single task	1.685	0.473	0.597	–
	Dual task	1.381	1.677	0.218	–
Anterior trunk flexion/camptocormia with thoracic fulcrum	Single task	2.208	1.199	0.317	–
	Dual task	1.704	0.253	0.743	–
Anterior trunk flexion/camptocormia with lumbar fulcrum	Single task	1.541	4.811	**0.025**	T0 vs. 1 min, *P*: 0.302 T0 vs. 2 min, *P*: 0.227 T0 vs. 3 min, *P*: 0.132
	Dual task	1.443	8.763	**0.004**	T0 vs. 1 min, *P*: **0.048** T0 vs. 2 min, *P*: **0.030** T0 vs. 3 min, *P*: **0.038**

Values in bold font indicate statistically significant difference.

*P*‐values refer to one‐way ANOVA (analysis of variance) analysis, with post hoc pairwise comparisons.

### Explorative Comparison between Different Tasks and Dual‐Task Interference

The comparison of single‐task versus dual‐task prolonged standing showed significant differences in all the postural angles, with worse ATF at the thoracic fulcrum (*P* < 0.001), ATF at the lumbar fulcrum (*P*: 0.002), and LTF (*P*: 0.027) during d.standing. The comparison of single‐task versus dual‐task walking showed a trend of worsening of the ATF at the lumbar fulcrum during the performance of the d.walking when compared to the s.walking, albeit not reaching the significant statistical threshold (*P*: 0.088). No other postural angles showed significantly different behavior comparing single‐task and dual‐task walking (Table 4).

**TABLE 4:** Mean and standard deviations of the degree of trunk bending during single and dual tasks while standing and walking

Minute	Single‐task standing				Dual‐task standing				Single‐task walking				Dual‐task walking			
	0	1	2	3	0	1	2	3	0	1	2	3	0	1	2	3
ATF‐t	42 (14.9)	41 (12.5)	41.5 (21.1)	43.9 (21.6)	42 (14.9)	49.4 (17.2)	48.7 (18.1)	48.7 (18.9)	41.2 (10.5)	40.4 (11.5)	43.2 (9.5)	43.4 (9.4)	41.2 (10.5)	40.2 (11.5)	41.6 (11)	41.6 (12.3)
ATF‐l	28.5 (17.3)	32 (20.6)	30.2 (18.7)	31.8 (18.6)	28.5 (17.3)	39.3 (21.8)	39.4 (23.1)	40.8 (23.8)	31.1 (16.9)	35.6 (15.8)	36.2 (17.5)	37.2 (19.1)	31.1 (16.9)	37 (16)	37.6 (17.6)	38.3 (17.9)
LTF	4.9 (4.6)	6.3 (6.3)	7 (6.8)	6.8 (6.2)	4.9 (4.6)	12.7 (12.1)	12.1 (13.1)	11.5 (12.6)	7.1 (6.7)	5.9 (4.9)	6.1 (4.4)	7.7 (7.4)	7.1 (6.7)	8.7 (7.6)	6.9 (5.6)	8 (5.9)

Data are expressed as mean degrees (standard deviation).

Abbreviations: ATF‐t, anterior trunk flexion at thoracic fulcrum; ATF‐l, anterior trunk flexion at lumbar fulcrum; LTF, lateral trunk flexion.

Comparing the s.standing with the s.walking tasks, we observed a significantly worse angle in the ATF at the lumbar fulcrum while walking (*P*: 0.019). No significant difference appeared in the ATF at the thoracic fulcrum and in the LTF between the s.standing and the s.walking tasks. Comparing the d.standing with the d.walking tasks, we observed a significantly worse angle in the ATF at the thoracic fulcrum while standing (*P*: 0.019), in the absence of significant differences in the ATF at the lumbar fulcrum and in the LTF (Table 4).

**FIG. 4:**
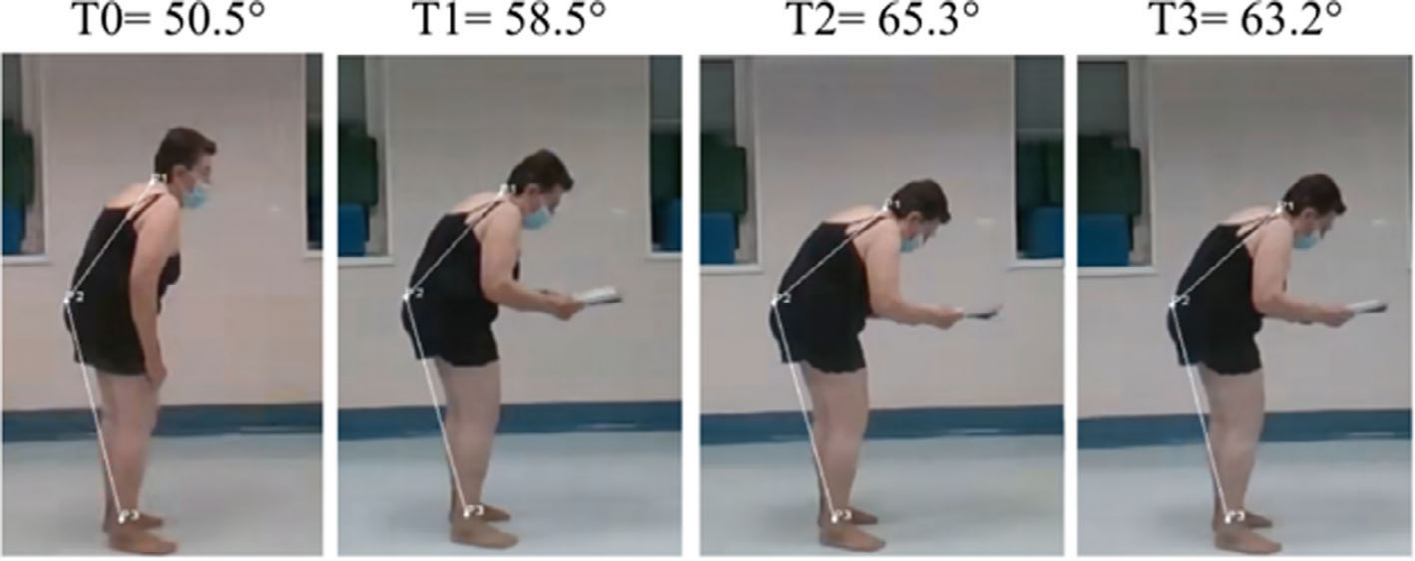
An example of the change in posture (anterior trunk flexion) over time while standing during a reading task.

We observed a dual‐task interference in the degree of trunk flexion after 3 min of static standing in the ATF thoracic (−18%) and lumbar (−28.3%) fulcrum and LTF (−70%). We found a dual‐task interference in the degree of trunk flexion after 3 min of walking standing in the ATF lower (−2.9%) and LTF (−4.3%) (Fig. 4).

### Correlation between Motor and Balance Impairment and Changes in Posture

We found a significant direct correlation between the severity of motor impairment (MDS‐UPDRS, Part III, score) and the change in the ATF at the thoracic fulcrum during d.standing (r: 0.66, *P*: 0.006). No other correlations were observed during the different tasks with other postural angle changes, nor were correlations found with Mini‐BESTest.

## Discussion

In this study, we proved the feasibility and usefulness of dynamic recording of APAs in PD using ad hoc software and 2 cameras for automatic analysis of posture. The AutoPosturePD enabled the assessment of the angle of trunk flexion both during static and dynamic and during single (motor) task and dual (motor–cognitive) task conditions. In addition, the analysis of data acquired by this first study allowed us to draw some interesting findings regarding the behavior of APAs in different dynamic conditions, which is still an unexplored research area. We observed different behaviors of postural angles analyzed depending on the task. Specifically, we found a significant worsening of the degree of LTF during prolonged standing, under both single‐ and dual‐task conditions; conversely, ATF (both at the lumbar fulcrum and at the thoracic fulcrum) showed significant changes only while performing the dual task. During walking, only patients with ATF with lumbar fulcrum exhibited a significant worsening of the degree over 1, 2, and 3 min under dual‐task condition. These results suggest that 1 min of static standing while reading may be sufficient to obtain a reliable degree of ATF and LTF.

The pathophysiology of APAs in PD still needs to be elucidated, and several causes may concur with their onset and worsening, such as central mechanisms (eg, imbalance in basal ganglia functioning leading to dystonia/rigidity, altered sensory–motor integration, higher cognitive function deficits) or peripheral mechanisms (ie, alteration in the musculoskeletal system).^3^, ^16^ It is largely known that PD patients may often have dual‐task difficulties involving both motor and cognitive functions,^17^ and the presence of APAs adds an additional challenge. APAs proved to be a source of disability during daily‐life activities of PD patients, leading to a worse quality of life. Several actions during daily life have the potential for negatively affecting the posture of PD patients, as activities like talking, counting, and walking compete for similar cognitive resources, and may lead to the so‐called “cognitive‐motor interference.”^7^, ^9^, ^10^, ^11^ PD patients, in particular, show more severe motor impairment in the motor–cognitive dual‐task condition, and this specific issue can impair the ability to keep the right posture. It is difficult to explain the reasons why ATF and LTF may exhibit different behaviors under dual‐task standing and walking, as observed in our study; interestingly, there are some clues from the literature supporting different pathophysiology underpinnings not only between LTF and ATF but also between ATF at the thoracic fulcrum and ATF at the lumbar fulcrum.^4^, ^18^ One possible explanation to the observation that posture tends to worsen more during prolonged standing than walking while performing a dual task is that the motor task of walking might positively influence postural correction by directing attention to movement (also considering the frequent direction changes while walking back and forth), whereas prolonged standing might lead to a decline in attention. This observation suggests that dynamic postural worsening in PD is more related to attentional and visuospatial control rather than fatigue.^19^

The comparison of single‐task versus dual‐task prolonged standing showed significant differences in all the postural angles, with worse ATF at the thoracic fulcrum, ATF at the lumbar fulcrum, and LTF during dual‐task standing. Comparing the single‐task standing with the single‐task walking, we observed a significantly worse angle in the ATF at the lumbar fulcrum while walking.

Noteworthy, MDS‐UPDRS, Part III, or Mini‐BESTest did not correlate with changes in postural angles during prolonged standing or walking, with the exception of ATF at the thoracic fulcrum. In fact, ATF worsening during 3 min of motor–cognitive task showed a correlation with higher MDS‐UPDRS, Part III, scores, suggesting that walking while performing a cognitive task may interfere with the ability to keep the trunk straight at a thoracic level. The consequences are limitations in mobility, reduced autonomy in real‐life settings, and increased risk of falling.

Overall, we believe that these findings hold significant implications for clinical practice. Image‐based measurements may not accurately detect the true degree angle because APAs may appear or get worse under prolonged/dynamic conditions, such as during walking or under dual‐task situations, resulting in different degrees of severity when compared with the static assessment. Therefore, professionals such as movement disorders experts, neurologists, physiatrists, and physiotherapists working with PD patients require a reliable, easy‐to‐use, and time‐efficient tool for the early detection, diagnosis, and monitoring of APAs. To the best of our knowledge, only 1 study was recently published evaluating the effectiveness and validity of the subjectively rated 2‐min standing endurance test for capturing temporal changes in APAs in patients with PD.^8^ The authors demonstrated some changes in the ATF with lower fulcrum over time after 2 min; however, they did not determine temporal angle changes for ATF with thoracic fulcrum using the upper camptocormia method, which is the most frequently reported in PD.^2^, ^4^

Our study showed that AutoPosturePD can detect the degree of postural change in patients with different PD severity, even those supported by a walker, and includes both the LTF and ATF with upper and lower fulcrum. Moreover, differently from the previous study,^8^ we demonstrated that less time is necessary—1 min during dual task— to objectively detect the real degree of APAs.

Such a monitoring is relevant for different reasons: (1) better characterization of the “natural history” of APAs in PD, (2) detection of changes resulting from pharmacological and nonpharmacological (ie, physical therapy) interventions, and (3) early detection of mild forms of ATF or LTF to provide recommendation and treatments possibly impacting their evolution in severe forms like camptocormia and Pisa syndrome. Therefore, the possibility to easily assess posture in a more reliable and ecologically valid way (eg, in dynamic condition and with the interference of cognitive performances) can offer a useful source of information for research and clinical practice.

Our study is not without technical limitations, which may reduce the applicability of the AutoPosturePD in clinical practice. Possible measurement errors can occur mainly due to (1) inadequate clinical setting, (2) poor environmental light, (3) improper camera position, or (4) inadequate chromatic contrast between the color of patient's skin/underwear and the background, as already reported in previous studies.^5^, ^6^ Therefore, it is important to get the full image of the patients, possibly without clothes, and with a correct height and distance between the camera and the patient. Moreover, not every place is suitable for taking the image because a certain degree of chromatic contrast is needed for the maximum reliability of the assessment. The technical artifact of image distortion, which could possibly introduce a bias in reliable angle calculation, was limited by the adequate distance of cameras from the patient's path and by the use of data extraction during walking tasks of time frames, including the proper alignment of patients and cameras (see Supplementary Material S1; Fig. 1). We did not encounter technical issues with the use of the software and, for each patient, an investigator made revision of angles calculated by the software using a visual inspection of videos and, in cases of doubts, compared the automatic angles of frames with the standard angle calculation based on images.^2^ No signs of inaccuracy in angle calculation were found, also considering 4 patients presenting mild limb dyskinesia.

Regarding the clues obtained by the postural behavior of patients, it is important to highlight that we have included a small sample of patient videos obtained by a center with experience in movement disorders and only on camptocormia and Pisa syndrome, excluding antecollis, another invalidating APAs. A wider, multicenter validation is therefore recommended before the implementation of the AutoPosturePD into clinical practice. Another aspect to be taken into account for the generalizability of the results is the use of a fixed sequence of tasks, which might influence the between‐task comparison due to fatigue. Moreover, we did not perform a formal neuropsychological assessment, which could be relevant especially considering the influence of cognitive tasks on posture. Finally, some patients used a walking aid during the tasks. This aspect can be interpreted as strength of the work for its real‐life connotation and the demonstration of trunk posture measurement also in patients using walking aids; however, it can somehow affect the posture evaluation, and this is something to be verified in future studies. All these possible biases should be considered in future studies using videos to assess posture under dynamic conditions in PD.

These limitations notwithstanding, these results suggest that 1 min of static standing while reading may be sufficient to obtain a true degree of LTF and ATF. The ATF at the lumbar fulcrum seems the only APAs for which a walking test with a cognitive dual task should be necessary to reliably disclose the severity of trunk flexion.

Regarding the potential future applicability of AutoPosturePD in clinical studies (including trials) and in clinical practice, it is important to highlight that this technique currently has some technical constraints. These include the requirement of an adequate room (spacious and with appropriate lighting and background conditions) and the proper positioning of 2 cameras, as well as technical expertise and resources to run the software (ie, adequate computer specifications and sufficient hard disk space for video storage), which can also be time consuming. Improvements in the software and validation of patient assessments (eg, requiring only 1 min of standing to capture the most clinically meaningful severity of APAs) could simplify the process and make the technique more feasible for widespread adoption in both research and clinical practice.

In conclusion, we believe that the new software presented in this study has the potential to become an easy‐to‐use tool fostering uniformity in the assessment of APAs in research studies and also in clinical practice to use under the dynamic conditions to assist health carers involved in the assessment and management of PD for the early detection, diagnosis, and management of APAs, which deserve prompt management interventions.

## Author Roles

All authors have contributed to the research and/or article preparation. All authors have approved the final version of the manuscript. This article represents the original work of the authors, has not been published elsewhere, and is not under consideration for publication elsewhere.

(1) Research project: A. Conception, B. Organization, C. Execution; (2) Statistical analysis: A. Design, B. Execution, C. Review and critique; (3) Manuscript preparation: A. Writing of the first draft, B. Review and critique.

C.A.A.: 1A, 1C, 2A, 2B, 3A

C.G.: 1B, 1C, 2A, 2C, 1A

C.P.: 1C, 2C, 3A

S.C.: 1B, 1C, 3B

S.A.: 1C, 2C, 3B

L.L.: 2B, 3B

M.T.: 1A, 1B, 2C, 3B

N.B.: 1A, 1B, 2C, 3B

## Disclosures

**Ethical Compliance Statement**: The patients provided written consent to participate in the study. We confirm that we have read the journal's position on issues involved in ethical publication and affirm that this work is consistent with those guidelines. The Verona Institutional Review Board approved the study (1655CESC).

**Funding Sources and Conflicts of Interest**: Carlo Alberto Artusi received speaker honoraria from AbbVie, Bial, Zambon, and Lusofarmaco. Christian Geroin reports no financial disclosures. Clarissa Pandino reports no financial disclosures. Serena Camozzi reports no financial disclosures. Stefano Aldegheri reports no financial disclosures. Leonardo Lopiano reports speaker honoraria from UCB, Bial, AbbVie, and Zambon. Michele Tinazzi reports no financial disclosures. Nicola Bombieri reports no financial disclosures. Financial support for the study was provided by the Fondazione Giuseppe Manni per la ricerca nelle neuroscience E.T.S. The authors declare that there are no conflicts of interest relevant to this work.

**Financial Disclosures for the Previous 12 Months**: The authors declare that there are no additional disclosures to report.

## Supporting information


**Data S1.** Supplementary information.

## Data Availability

The data that support the findings of this study are available from the corresponding author upon reasonable request.

## References

[1] Tinazzi M , Gandolfi M , Ceravolo R , et al. Postural abnormalities in Parkinson's disease: an epidemiological and clinical multicenter study. Mov Disord Clin Pract 2019;6(7):576–585. 31538092 10.1002/mdc3.12810 PMC6749805

[2] Tinazzi M , Geroin C , Bhidayasiri R , et al. Task force consensus on nosology and cut‐off values for axial postural abnormalities in parkinsonism. Mov Disord Clin Pract 2022;9(5):594–603. 35844289 10.1002/mdc3.13460 PMC9274349

[3] Geroin C , Artusi CA , Nonnekes J , et al. Axial postural abnormalities in parkinsonism: gaps in predictors, pathophysiology, and management. Mov Disord 2023;38:732–739. 37081741 10.1002/mds.29377

[4] Margraf NG , Wolke R , Granert O , et al. Consensus for the measurement of the camptocormia angle in the standing patient. Parkinsonism Relat Disord 2018;52:1–5. 29907329 10.1016/j.parkreldis.2018.06.013

[5] Artusi CA , Geroin C , Imbalzano G , et al. Assessment of axial postural abnormalities in parkinsonism: automatic picture analysis software. Mov Disord Clin Pract 2023;10(4):636–645. 37070056 10.1002/mdc3.13692 PMC10105105

[6] Aldegheri S , Artusi CA , Camozzi S , et al. Camera‐ and viewpoint‐agnostic evaluation of axial postural abnormalities in people with Parkinson's disease through augmented human pose estimation. Sensors 2023;23:3193. 36991904 10.3390/s23063193 PMC10058715

[7] Kelly VE , Eusterbrock AJ , Shumway‐Cook A . A review of dual‐task walking deficits in people with Parkinson's disease: motor and cognitive contributions, mechanisms, and clinical implications. Parkinsons Dis 2012;2012:918719. 22135764 10.1155/2012/918719 PMC3205740

[8] Kondo Y , Ariake Y , Suzuki I , et al. Two‐minute standing endurance test for axial postural abnormalities in patients with Parkinson's disease. Gait Posture 2024;112:81–87. 38749293 10.1016/j.gaitpost.2024.05.001

[9] Plummer P , Zukowski LA , Giuliani C , Hall AM , Zurakowski D . Effects of physical exercise interventions on gait‐related dual‐task interference in older adults: a systematic review and meta‐analysis. Gerontology 2015;62:94–117. 25721432 10.1159/000371577

[10] Snijders AH , Verstappen CC , Munneke M , Bloem BR . Assessing the interplay between cognition and gait in the clinical setting. J Neural Transm 2007;114:1315–1321. 17612789 10.1007/s00702-007-0781-x

[11] Kelly VE , Janke AA , Shumway‐Cook A . Effects of instructed focus and task difficulty on concurrent walking and cognitive task performance in healthy young adults. Exp Brain Res 2010;207:65–73. 20931180 10.1007/s00221-010-2429-6 PMC3058115

[12] Bloem BR , Valkenburg VV , Slabbekoorn M , Willemsen MD . The multiple tasks test: development and normal strategies. Gait Posture 2001;14:191–202. 11600322 10.1016/s0966-6362(01)00141-2

[13] Postuma RB , Berg D , Stern M , et al. MDS clinical diagnostic criteria for Parkinson's disease. Mov Disord 2015;30:1591–1601. 26474316 10.1002/mds.26424

[14] Stebbins GT , Goetz CG , Burn DJ , Jankovic J , Khoo TK , Tilley BC . How to identify tremor dominant and postural instability/gait difficulty groups with the movement disorder society unified Parkinson's disease rating scale: comparison with the unified Parkinson's disease rating scale. Mov Disord 2013;28:668–670. 23408503 10.1002/mds.25383

[15] Li KZH , Bherer L , Mirelman A , Maidan I , Hausdorff JM . Cognitive involvement in balance, gait and dual‐tasking in aging: a focused review from a neuroscience of aging perspective. Front Neurol 2018;9:913. 30425679 10.3389/fneur.2018.00913 PMC6219267

[16] Artusi CA , Geroin C , Nonnekes J , et al. Predictors and pathophysiology of axial postural abnormalities in parkinsonism: a scoping review. Mov Disord Clin Pract 2023;10(11):1585–1596. 38026508 10.1002/mdc3.13879 PMC10654876

[17] McIsaac TL , Fritz NE , Quinn L , Muratori LM . Cognitive‐motor interference in neurodegenerative disease: a narrative review and implications for clinical management. Front Psychol 2018;9:2061. 30425673 10.3389/fpsyg.2018.02061 PMC6218850

[18] Doherty KM , van de Warrenburg BP , Peralta MC , Silveira‐Moriyama L , Azulay JP , Gershanik OS , Bloem BR . Postural deformities in Parkinson's disease. Lancet Neurol 2011;10:538–549. 21514890 10.1016/S1474-4422(11)70067-9

[19] Artusi CA , Montanaro E , Erro R , et al. Visuospatial deficits are associated with Pisa syndrome and not camptocormia in Parkinson's disease. Mov Disord Clin Pract 2022;10(1):64–73. 36704069 10.1002/mdc3.13605 PMC9847315

