# Supplementary material for: Dynamic Video Assessment of Axial Postural Abnormalities in Parkinson's Disease: A Pilot Study
Source: Mov Disord Clin Pract. 2025 Jan 29;12(5):626–37. doi: 10.1002/mdc3.14329 (PMC12070167; doi:10.1002/mdc3.14329)
Supplement: Supplementary file 1 — Data S1. Supplementary information. [file MDC3-12-626-s001.docx]

**Supplementary material - Detailed description of tasks and measurements**

For the dynamic analysis of posture, each patient performed four different tasks, each lasting 3 minutes. Assessments were conducted in a well-artificially-lit large room (with natural light excluded by closing nearby windows) measuring approximately at least 7 meters in length and 4.5 meters in width. This setup provided a 6-meter walking path and space for positioning two cameras for continuous real-time recording. These cameras connected to two laptops captured the full figure of each patient from both coronal and sagittal views during standing and walking tasks. The camera recoding the sagittal view is positioned horizontally while camera recording the frontal view is positioned vertically, both on a stable surface. Since patients may walk having assistance devices such as canes or walkers, two rectangular areas (1.5 x 1 meters) were designed at the beginning and at the end of walking path to accommodate turning maneuvers (Figure 1).

The experimental conditions were as follows:

1. The patient stood still at a central point in the room (about 2.30m distance from the coronal view camera, and 3.30m distance from the sagittal view camera), aligned with the right shoulder toward the camera focus recoding the sagittal view, for 3 minutes in a quiet condition, instructed to remain motionless without performing any voluntary tasks or speaking.
2. The patient stood still in the same central position for 3 minutes while reading aloud a text and instructed to stay motionless.
3. The patient walked back and forth at a self-selected comfortable speed along a lane in the center of the room, aligned with the camera focus, for 3 minutes, starting from the center of the room, without speaking or performing other tasks. At the beginning and end of walking task, the patient was required to keep still standing position for at least 5 seconds.
4. The patient walked back and forth at a self-selected comfortable speed along the same lane, starting from the center of the room, for 3 minutes while instructed to count aloud backward from 100 to 0 in decrements of 7, focusing primarily on the countdown. At the beginning and end of walking task, the patient was required to keep still standing position for at least 5 seconds.

During all four tasks, continuous video recordings were taken from the two RGB-D cameras positioned to capture the patient's full posture: for the sagittal view in landscape mode at waist height, approximately 1.05 meters from the ground, while for the coronal the camera was set to portrait at 0.70 meters from the ground. Trunk flexion angles were automatically calculated by software, measuring the angle of anterior trunk flexion at the thoracic fulcrum, anterior trunk flexion at the lumbar fulcrum, and lateral trunk flexion. These measurements were based on the criteria defined by the MDS Task Force on Postural Abnormalities in Parkinsonism^1^ and using a validated algorithm for automatic posture analysis from images.^2,3^

In brief, a state-of-the-art Human Pose Estimation (HPE) platform based on Deep Neural Networks (DNNs), enhanced with specific key features, was used to obtain real-time measurements of spinal flexion angles and fulcra in accordance with MDS Task Force criteria.^1^ This was achieved through a post-processing software application that, using key-point data from the HPE software, automatically identifies anatomical landmarks: the last spinous process of the cervical vertebra (C7), the last spinous process of the lumbar vertebra (L5), the midpoint between the ankles (MA) for both frontal and sagittal views, and the point (fulcrum, FC) farthest from the line between C7 and L5 in the sagittal view.^2^

Using the software, we obtained continuous values for the angles of anterior trunk flexion at the thoracic fulcrum, anterior trunk flexion at the lumbar fulcrum, and lateral trunk flexion from the video recordings at any given time point during each 3-minute task. From the 3-minute of data gathering of each task, we extracted continuous measurement of the degree of trunk flexion at the beginning of the task and after 1, 2, and 3 minutes. For the walking tasks, measurements were analyzed only during the timeframes in which the patient was aligned with the focus of the camera, within a focal range of approximately 1.5 meters (see the yellow triangle in Figure 1).

This approach was implemented to enhance the accuracy of angle calculations, reducing significant distortion and preventing potential misinterpretation of angles.^4^

Finally, to avoid possible measurement errors caused by inadequate chromatic contrast between the patient's skin/underwear and the background, patients were asked to wear underwear in a color different from that of the room's walls.^2,3^

**References for supplementary methods**

1. Tinazzi M, Geroin C, Bhidayasiri R, et al. Task Force Consensus on Nosology and Cut-Off Values for Axial Postural Abnormalities in Parkinsonism. Mov Disord Clin Pract 2022;9:594-603.
2. Artusi CA, Geroin C, Imbalzano G, et al. Assessment of Axial Postural Abnormalities in Parkinsonism: Automatic Picture Analysis Software. Mov Disord Clin Pract 2023;10:636-645.
3. Aldegheri S, Artusi CA, Camozzi S, et al. Camera- and Viewpoint-Agnostic Evaluation of Axial Postural Abnormalities in People with Parkinson's Disease through Augmented Human Pose Estimation. Sensors 2023;23:3193.
4. Boldo M, Di Marco R, Martini E, et al. On the reliability of single-camera markerless systems for overground gait monitoring. Comput Biol Med 2024;171:108101.
